# Exploring the experiences of nurses caring for patients with COVID-19: a qualitative study in Iran

**DOI:** 10.1186/s12912-022-00805-5

**Published:** 2022-01-14

**Authors:** Sudabeh Ahmadidarrehsima, Nasibeh Salari, Neda Dastyar, Foozieh Rafati

**Affiliations:** 1 Department of Nursing, Nursing and Midwifery School, Jiroft University of Medical Sciences, Jiroft, Iran; 2 Department of Midwifery, Nursing and Midwifery School, Jiroft University of Medical Sciences, PO Box:7861763730, Jiroft, Iran

**Keywords:** Qualitative research, Experiences, Nurses, COVID-19

## Abstract

**Background:**

The coronavirus disease (COVID-19) is now a major public health emergency in the world. Nurses as key members of the COVID-19 patient care team are exposed to most challenges caused by the disease. As exploring the experiences of nurses as patient supporters and caregivers can play an important role in improving the quality of care for patients with COVID-19 disease, the present study explored the experiences of nurses caring for patients with COVID-19.

**Methods:**

The study employed a qualitative design. This study employed purposive sampling to select 10 nurses with bachelors and master’s degrees in nursing who were taking care of patients with COVID-19 in ICUs or inpatient wards in southern Iran. Data were collected through semi-structured interviews. The collected data were analyzed using the qualitative content analysis procedure proposed by Graneheim and Lundman.

**Results:**

The analysis of the data revealed four main themes and ten sub-themes: A) physical, psychological, and social burden of care (excessive workload; fear, anxiety, worry; unpleasant social experiences; compassion fatigue) B) unmet needs (personal needs and professional needs) C) positive experiences (pleasant social experiences and inner satisfaction), and D) strategies (problem-solving strategies and stress symptom mitigation strategies).

**Conclusions:**

An analysis of the themes and subthemes extracted in this study suggested that the nurses who participated in this study faced many personal and professional challenges. Therefore, health officials and specialists need to pay special attention to nurses’ challenges and needs.

## Introduction

COVID-19 disease, caused by the coronavirus, was first diagnosed in December 2019 in people with lung infections in Wuhan, China [1]. The disease has a variety of symptoms ranging from mild symptoms to pneumonia, severe shortness of breath, septic shock, and even failure in various organs of the body [2]. No specific treatment has yet been discovered for this disease, and only some antiviral drugs or analgesics and antipyretics are used to alleviate its symptoms [3]. The prevalence of COVID-19 disease is a major public health problem worldwide. On March 11, 2020, the World Health Organization declared the disease a pandemic [4]. Although the world has witnessed many infectious diseases in the past, the outbreak of COVID-19 disease as a new infectious disease has severely challenged the health systems of many countries [5]. The disease has had a high incidence rate in different countries of the world. Reports from the Worldometer database show that for instance, up to September 27, 2020, the total number of confirmed COVID-19 cases was 33, 58,557 persons with a total of 998,747 deaths, and 24,411,621 treated patients in the world, According to the official statistics of the Ministry of Health and Medical Education of Iran, until September 27, 2020, the number of patients with COVID-19 had reached 443, 86 persons and a total of 25,394 people had lost their lives [6].

During the outbreak of infectious diseases, health care and medical staff always play the most important role in treating patients, and thus their health is endangered or they may even lose their lives [7] Also, as the largest group of health professionals, nurses work at the frontline of the healthcare system and provide direct care to patients with COVID-19 disease [8]. Long work hours and high workload, exposure to the virus, frequent and close contact with patients infected with COVID-19, the lack of personal protective equipment (PPE), stigmatization as a vector of the virus, media pressure, and increased number of deaths could lead to psychological distress and exhaustion in them [8–10]. Furthermore, nonstandard PPE, excessive sweating and dehydration, wounds and the feeling of suffocation caused by wearing masks for a long time, and lack of proper conditions for eating and drinking could lead to further physical burden in nurses during the COVID-19 pandemic [10]. Such factors cause feelings of hopelessness and helplessness in nurses and reduce the quantity and quality of patient care [11, 12].

Explaining nurses’ perception of caring for patients with COVID-19 may be helpful for improving and promoting the health of patients [13]. As qualitative research is often used to explore phenomena in depth [14], conducting a study with a qualitative approach will lead to a better understanding of nurses’ experience of caring for patients with COVID-19 disease. Due to limited studies on the experiences of nurses working in COVID-19 wards in Iran, the present study was conducted to provide a clear picture of the care experiences of Iranian nurses during the COVID-19 outbreak. Also, nurses may employ the insights offered in this study in practicing effective self-care strategies.

## Materials and methods

### Design and participants

This qualitative study was conducted with a conventional content analysis approach to explore nurses’ experience of caring for COVID-19 patients in the south of Iran. The research population was nurses working in the wards and intensive care units (ICU) where patients with COVID − 19 were cared for. The inclusion criteria were having at least four weeks of experience in caring for COVID-19 patients, having at least a bachelor’s degree in nursing, and willingness to participate in the study The participants were selected using the purposive sampling method based on the duration of work as a nurse, the length of experience in the COVID-19 ward, age, and gender. Also, to ensure the diversity of the data, nurses caring for patients with COVID-19 both in the intensive care unit and the inpatient wards were interviewed.

### Data collection

The data were collected via face-to-face in-depth semi-structured interviews from December 2020 to February 2021. First, the names and contact information of the nurses who worked in the COVID-19 care units (ICU and inpatient wards) were obtained from the nursing office. The nurses were then contacted and the goals and methods of the study were explained to them, and after obtaining oral consent from the nurses who were willing to participate in the study, the time and place of the interviews were arranged. All interviews were conducted in the hospital upon the participants’ agreement and recorded by a digital recorder. The interviews were conducted using an interview guide including some key open-ended questions that allowed participants to express their experiences as fully as possible. The guide contained questions such as: “Please tell me about your experience in caring for a hospitalized patient with confirmed COVID-19”. Following the guide questions and according to the participant answers, follow-up questions were used to address all aspects of a part of the topic. Probing questions such as “Can you please explain more?” and “Can you please give an example?” were used where necessary. Sampling continued until data saturation. Data saturation occurred after the interview with 8 nurses. To ensure that there was no other new data, the researchers conducted two more interviews. Two nurses needed to be interviewed again for clarifying their experiences. As a result, the number of interviews increased to 12 with 10 nurses. Each interview lasted from 40 to 60 min.

### Data analysis

Data collection and analysis were performed simultaneously. The data were analyzed using Graneheim and Lundman’s content analysis approach [15]. First, each interview was transcribed verbatim. Then, the texts were read several times to understand the meanings related to participants’ experiences. In the second step, the initial codes were extracted based on meaning units. Then, the related codes were merged according to their similarities and differences and main and sub-categories were formed. Finally, the hidden concepts emerged from the data. MAXQDA 12 software was used to manage the data.

### Rigor

The rigor of the study was tested using Guba and Lincoln’s criteria [16]. Credibility was obtained through the researcher’s long-term involvement with the participants and providing the initial codes to the participants for reviewing and confirming them. To ensure dependability, the study and data analysis process was carefully performed by the research team. For this purpose, the two authors (FR and SAD) performed the coding of the texts independently and then met to discuss any disagreements and achieve consensus about initial coding and emerged categories. To evaluate the confirmability, two faculty members specializing in qualitative research confirmed the data analysis process. Maximum diversity sampling, rich description of findings, and confirmation of findings by two non-participating nurses helped to ensure data transferability.

### Ethical considerations

This project was approved by the ethics committee of Jiroft University of Medical Sciences (IR.JMU.REC.1399.031). Before obtaining informed consent, the necessary explanations about voluntary participation, anonymity, confidentiality, and safe protection of the data were given to participants. In addition, the participants were informed that they could leave the study at any time.

## Results

In this study, ten nurses who worked in COVID-19 and ICU COVID-19 wards were interviewed. Four participants were male and the rest were female (Table 1).

**Table 1:** The participants’ demographic characteristics

Participant’s code	Age (year)	Sex	work experience (Years)	Marital Status	Level of Education	Ward	Position
1	29	female	5	Single	Bachelor of degree	COVID	Nurse
2	25	female	3	Married	Master of degree	COVID	Nurse
3	25	female	2	Single	Bachelor of degree	COVID	Nurse
4	44	female	15	Married	Bachelor of degree	COVID	Head Nurse
5	30	female	7	Single	Bachelor of degree	ICU COVID	Nurse
6	32	female	5	Married	Bachelor of degree	ICU COVID	Nurse
7	25	Male	3	Single	Bachelor of degree	COVID	Nurse
8	37	Male	11	Married	Master of degree	ICU COVID	Nurse
9	41	Male	13	Married	Bachelor of degree	ICU COVID	Head Nurse
10	35	Male	9	Married	Bachelor of degree	ICU COVID	Nurse

Analysis of the data revealed 4 main themes: (1) Physical, psychological, and social burden of care, (2) Unmet needs, (3) Positive experiences, and (4) Strategies. (Table 2).

**Table 2:** The themes and sub-themes extracted after data analysis

Main Themes	Sub-Themes
Physical, Psychological, and Social burden of care	excessive workload; fear, anxiety, worry; unpleasant social experiences; and compassion fatigue
Unmet Needs	personal needs and professional needs
Positive Experiences	pleasant social experiences and inner satisfaction
Strategies	problem-solving strategies and stress symptom mitigation strategies

Table 2: The main themes and sub-themes that emerged from the data collected from nurses^’^ experiences in caring for patients with COVID-19 disease.

### The physical, psychological and social burden of care

This theme refers to the physical, psychological, and social problems experienced by the nurses.

#### Excessive workload

All the participants in this study pointed to the difficulties of working in the COVID-19 ward. Excessive workload, hard work, feeling of extreme heat due to wearing protective clothing, thirst, skin problems due to excessive sweating and wearing a mask, difficulty in going to the toilet, and strict compliance with protective protocols were some of the problems reported by the participants. They stated that these problems put them under a lot of work pressure and they became exhausted. For instance, one of the participants said, “*From the onset of the shift and entering the ward, we could not rest even for a moment ... It was terrible. I felt I was taking part in a war and I was fighting*” (Participants No. 4).

#### Fear, anxiety, and worry

The participants stated that various issues made them feel afraid and anxious. This anxiety persisted not only at work but also outside the workplace. It was so serious that in some cases it led to the participants’ irritation and alertness to hear the news about the transmission of the disease by them to family members. The most important causes of the nurses’ fear and anxiety were the risk of COVID-19 infection, the possibility of transmitting the disease to family members, sudden deterioration of some patients’ conditions, high mortality rates, preoccupation with the possibility of a low engagement or not making enough effort to save patients, and knowledge and skill inadequacy to help critically-ill patients. “*I’m stressed for transmitting the disease to my family members, so that every time my phone is ringing, I get a heartbeat. I always fear that they call me and tell me they have the symptoms of the disease*” (Participant No. 5).

#### Compassion fatigue

The nurses stated that they suffered from mental distress due to exposure to anxiety, fear, suffering, family rejection, and patients’ death in isolation: “*the nurse’s worst experience can be a moment one of the patients takes your hand and says she is choking, or suddenly starts shivering or screaming in pain in the chest. The scenes bothered me a lot and made me nervous*” (Participant No. 2).

#### Unpleasant social experiences

Most of the nurses stated that they experienced the fear and the risk of being rejected by people or their families and described it as an unpleasant experience. They also considered the lack of empathy of colleagues in other wards and some cases the family’s dissatisfaction with their work in the ward for COVID-19 patients as unpleasant social experiences. One of the nurses said, “*When I left the ward, everyone was running away from me, even my relatives were staying away from me, and it was not pleasant*” (Participant No. 6).

### Unmet needs

These needs refer to personal and professional needs that were not met and made the nurses feel anxious.

#### Personal needs

The nurses stated that their physical and mental needs were neglected and they highlighted their need for receiving more psychological support. They also believed that in addition to the physical and mental needs, the need for job security which is important to every individual was not considered by the authorities because some unemployed nurses were hired under short-term employment contracts following the call of the Ministry of Health, and these nurses had no job security. They also needed to be appreciated by the authorities. The participants complained that the increase in their pays due to their work in the ward for patients with COVID was not commensurate with the difficulty of their work and was not a fair increase. As an example, one of the nurses said, “*Unfortunately, no one takes into account our mental and psychological needs. It would be good if the authorities paid attention to this issue as well*” (Participant No. 10).

#### Professional needs

According to the participants, lack of personal protection facilities and equipment, especially at the onset of the COVID-19 epidemic, lack of appropriate dressings for the treatment of pressure sores caused by constant wearing of masks, lack of nursing and service staff, lack of oxygen ventilators, and lack of proper ventilation in the ward were among the most common unmet professional needs reported by the nurses in this study. “*Early on when we opened the ward, we did not even have a service worker to disinfect the surfaces*” (Participant No. 2).

### Positive experiences

In addition to negative experiences and high workload, the nurses participating in this study also reported that they had pleasant and positive experiences. These experiences were divided into two subcategories:

#### Pleasant social experiences

These experiences refer to receiving positive feedback, support, and empathy from family members and people in the community. “*When I introduced myself as a nurse working in the COVID-19 ward, salesclerks gave me a discount, or people prayed for me or helped me if I needed help, and this created a good feeling in me*”. (Participant No. 1).

#### Internal satisfaction

In addition to receiving positive feedback from people, the participants also reported that they had internal and personal satisfaction. Some of them stated that a spiritual atmosphere prevailed in the ward and this spiritual atmosphere and self-sacrifice in the ward were enjoyable for them. All of the participants considered themselves almost national heroes and were proud of themselves. They also stated that the patients’ recovery made them feel happy, relieved of fatigue, and increased their motivation to continue working and caring for patients. According to the participants, it was because of these positive experiences that almost all of them were ready to work in conditions similar to this pandemic and believed that if they wanted to decide again to choose a workplace, they would still choose to work in the COVID-19 ward. “*One day one of our patients was discharged from the ward after a month. The patient and her son danced in front of the nursing station (to appreciate our services). The joy of seeing this scene is indescribable*”. (Participant No. 4).

### Strategies

The nurses stated that they used different strategies to cope with the stress of working in a high-mortality and busy ward:

#### Problem-solving strategies

Most of the nurses reported that they tried to find the root cause of the stress. For instance, they resorted to exercise and good nutrition to reduce stress caused by the risk of developing the disease. Besides, to relieve the stress caused by the possibility of transmitting the disease to others, they tried to maintain a physical distance from others and avoided contact with others. They also lived in a separate place to avoid any contact with other family members. Furthermore, they used a negotiation and persuasion strategy to reduce the stress caused by family members’ disagreement with their work in the COVID-19 ward. “*I equipped a room in the backyard to separate myself from my family members*”. (Participant No. 7).

#### Stress symptom mitigation strategies

The participants reported that in some cases, they used different strategies to reduce the symptoms of stress. These strategies were keeping their negative thoughts away, walking, reading books, using relaxation techniques, and reliance on God. Furthermore, adopting a professional approach to working in the COVID-19 ward and a sense of responsibility to help resolve the COVID-19 pandemic crisis also helped them to develop a rational attitude toward their work in the COVID-19 ward and reduce their anxiety. “*I was very scared that I might pass the disease on to my family members, but I relied on God and tried to keep those thoughts away from me*” (Participant No. 1).

## Discussion

The present study showed caring for COVID-19 patients was associated with a variety of experiences for nurses. An analysis of the nurses’ experiences revealed 4 main themes: [1] Physical, psychological, and social burden of care, [2] Unmet needs, [3] Positive experiences, and [4] Strategies. This section discusses these themes:

The participants in this study reported physical burden and fatigue due to excessive workload. Kackin et al. also, (2020) reported that caring for COVID-19 patients puts a heavy physical burden on nurses in Turkey [17]. Liu et al. (2020) also confirmed that caring for patients for long hours while wearing personal protective equipment (PPE) leads to nurses’ physical distress. In addition, physical distress became unbearable for nurses who had to remain in isolated wards [18]. Irandoost et al. (2020) also reported that challenges such as putting on and taking off personal protective equipment and limitations due to these clothes and equipment, fatigue, insomnia, headache, and anorexia led to excessive workload and physical and mental problems for nurses [19]. Karami et al. (2020) also pointed out that nurses working in COVID-19 wards had to endure unusual work difficulties to be able to provide real and high-quality care services [20]. In a similar vein, Sun et al. (2020) stated that self-protection acts caused discomfort, fatigue, and helplessness in Chinese nurses during the COVID-19 pandemic [21]. Thus nurses should be protected against excessive workload so that they can provide effective patient care.

In this study, findings showed that all participants experienced psychological symptoms such as fear, anxiety, and worry. These experiences may cause poor quality of life among nurses and can reduce the quality and quantity of patient care. Similarly, Karami et al. (2020) reported the feeling of fear, anxiety, and worry in the lived experiences of nurses caring for patients with COVID-19 in Iran. They emphasized that nurses working in the wards and care centers dedicated to COVID-19 patients experience poor mental, emotional, and occupational conditions, which jeopardize the provision of quality care [20]. Wu et al. (2019) attributed the feeling of fear, anxiety, and worry to uncertainty about the source of the virus, lack of specific treatment, high infection rate, the high mortality rate of healthcare workers, and fear of infection in nurses [22]. Zhang et al. (2020) reported that the reason for nurses’ fear, anxiety, and worry is the feeling of uncertainty about the COVID-19 situation and stated that these negative psychological feelings can lead to nurses’ stress or vulnerability [23]. Self-quarantine, isolation, children’s educational decline, fear of the future, PPE troubles, and hopelessness about treating patients are other reported sources of psychological burden in nurses during this pandemic [10]. Also, Kackin et al. (2020) suggested that caring for the patients, fear of infecting oneself and family members, and the stigma attached to nurses by society are some reasons behind nurses’ sense of fear, anxiety, and worry, making them obsessed and depressed [17]. To solve this problem, Chevance et al. (2020) recommended monitoring nurses’ psychological problems and implementing early interventions such as professional psychological counseling [24].

Another problem highlighted by the nurses participating in the present study was unpleasant social experiences. Nurses feel social isolation due to fear of being stigmatized by society and the risk of transmitting the disease [25]. Likewise, Kim et al. (2018) and Xiang et al. (2020) suggested that unpleasant social experiences have caused nurses to feel guilty and regretful, so they may prefer to limit their communication and live in a dormitory with no contact with others [26, 27]. Although health care workers, especially nurses, have been acknowledged as “health defenders” by the Iranian government and the people of Iran, their stigmatization as vectors of infection, which is a common phenomenon in the world, can be a source of stress and a reason for nurses’ social isolation and marginalization [28].

Also, in this study, findings showed that the nurses participating in the study had personal and professional needs that were not satisfied. These unsatisfied needs caused stress for them and could affect their patient care. It is necessary to pay attention to nurses’ personal and professional needs, try to satisfy their needs, and provide the facilities and equipment needed for patient care because, without effective protective equipment, nurses working with COVID-19 patients are directly exposed to serious risks [23]. Moreover, according to Maslow’s hierarchy of needs, the lowest and most basic human needs are physiological and safety needs [29]. Authorities and governments should not forget the physical and psychological burden imposed on nurses due to the COVID-19 epidemic, and they should pay attention to their physical, mental, and professional needs and take action to address it [30].

It should be noted that in addition to negative experiences, the nurses participating in this study had positive experiences that made the care of patients with COVID-19 enjoyable for them and created a positive inner feeling. Some of them reported that working in COVID-19 wards was a sacred activity. They also stated that enthusiasm and a sense of patriotism led them to work in the COVID-19 wards voluntarily. Wu et al. (2019) also suggested that when nurses witness the suffering of their patients, their sense of altruism and empathy compels them to work harder, and this gives them inner peace and satisfaction and encourages them to continue to move forward with courage and keep working [22]. Jenna et al. (2021) also showed that some nurses working in COVID-19 wards had positive feelings, such as pride and empowerment, towards working as a nurse during the COVID-19 pandemic [31]. Jing et al. (2020) also showed nurses had a spirit of selflessness towards patients, and this led to their inner peace [32].

The results of the present study showed that to cope with the stress of working in busy high-mortality COVID-19 wards, the nurses used different strategies including problem-solving and stress mitigation strategies. Most of the nurses tried to find the root cause of the stress and solve it. Also, they used different strategies to reduce the symptoms of stress. Accordingly, Irandoost et al. (2020) reported that nurses working in COVID-19 wards used strategies such as spiritualizing their work, trusting in God, engaging in religious activities, and creating an atmosphere of empathy to reduce work-related stress in the wards such as deprivation, mental health problems, fear, unpleasant experiences, and social and communication challenges [19] Also, nurses in Jenna et al.^’^s study tried to improve their self-care and reduce the stress caused by working during the COVID-19 pandemic with various strategies such as prayer, exercising, mindfulness, and virtual meetings with friends [31]. Zhang et al. (2020) found that nurses on the frontline fighting COVID disease − 19 positively taking measures to cope with stress, which is encouraging and indicates that most nurses managed to adapt to the situation by themselves [33].

## Conclusion

This study showed that nurses who were caring for COVID-19 patients faced many challenges including excessive workload, lack of protective equipment, psychological problems, fear, isolation in personal and family life, and lack of support in the workplace. To overcome these challenges, they used strategies such as solving the root cause of stress, doing religious and spiritual activities. They also tried to gain family support and create an empathetic work environment. Thus, some strategies can be adopted to reduce these challenges and provide better conditions for nurses including providing adequate protective equipment, providing a suitable work environment, financial and social support, effective management of work shifts, paying more attention to nurses’ physical and mental health, and developing appropriate mechanisms for nurses to communicate with their families, patients, patients’ family, and the community around them. Moreover, providing psychological counseling can help nurses overcome their psychological problems and improve their mental state.

### Limitations and strengths

Selecting nurses with maximum diversity to better understand their experiences of caring for patients with COVID-19 and conducting in-depth interviews to better understand nurses’ experiences were the strengths of this study. Although efforts were made to maximize the diversity of nurses, due to sample size limitations and the selection of nurses from one hospital, it cannot be guaranteed that all nurses’ experiences have been considered. The findings of this study may not be generalizable to nurses of other countries. Besides, despite informing participants about the data confidentiality, they did not disclose any unhealthy coping mechanisms that may be related to their embarrassment at disclosing such strategies.

## Data Availability

The datasets used and/or analyzed during the current study are available from the corresponding authors on reasonable request.

## Abbreviations

COVID-19: Corona Virus Disease of 2019
ICU: Intensive care unit
PPE: personal protective equipment

## References

[1] Stubinger J, Schneider L. Epidemiology of coronavirus COVID-19: Forecasting the future incidence in different countries. Healthcare. 2020;8(2):99. Multidisciplinary Digital Publishing Institute. 10.3390/healthcare8020099‏. 10.3390/healthcare8020099 PMC7349853 32326512

[2] Adhikari S (2020). Epidemiology, causes, clinical manifestation and diagnosis, prevention and control of COVID-19 disease during the early outbreak period: a scoping review. Infect Dis Poverty.

[3] Ahnd G (2020). Current status of epidemiology, diagnosis, therapeutics, and vaccines for novel COVID-19 disease 2019. J Microbiol Biotechnol.

[4] Bulut C (2020). Epidemiology of COVID-19. Turk J Med Sci.

[5] Zhu N, et al. A novel COVID-19 from patients with pneumonia in China, 2019. New Engl J Med. 2020. 10.1056/NEJMoa2001017 PMC7092803 31978945

[6] Asghari M (2021). The analysis of psychological experiences of the elderly in the pandemic of coronavirus disease: a phenomenological study. Aging Psychol.

[7] Hung L-SH (2003). The SARS epidemic in Hong Kong: what lessons have we learned?. J R Soc Med.

[8] Alvarez C, et al. COVID 19: psychological effects and associated factors in Mexican nurses. Disaster Med Public Health Preparedness. 2020:1–7. 10.1017/dmp.2020.495. 10.1017/dmp.2020.495 PMC7985626 33706845

[9] Carter W (2012). Recognition and referral of depression in patients with heart disease. Eur J Cardiovasc Nurs.

[10] Zamanzadeh V, Valizadeh L, Khajehgoodari M, Bagheriyeh F (2021). Nurses’ experiences during the COVID-19 pandemic in Iran: a qualitative study. BMC Nurs.

[11] Frawley T, van Gelderen F, Somanadhan S, Coveney K, Phelan A, Lynam-Loane P, de Brún A (2021). The impact of COVID-19 on health systems, mental health and the potential for nursing. Ir J Psychol Med.

[12] Liu C-Y, Yang YZ, Zhang XM, Xu X, Dou QL, Zhang WW, Cheng ASK (2020). The prevalence and influencing factors in anxiety in medical workers fighting COVID-19 in China: a cross-sectional survey. Epidemiol Infect.

[13] Galehdar N, Toulabi T, Kamran A, Heydari H (2021). Exploring nurses' perception of taking care of patients with coronavirus disease (COVID-19): a qualitative study. Nurs Open.

[14] Polit D-F, Beck C-T. Essentials of nursing research: appraising evidence for nursing practice: Lippincott Williams & Wilkins; 2013.

[15] Graneheim UH, Lundman B (2004). Qualitative content analysis in nursing research: concepts, procedures, and measures to achieve trustworthiness. Nurse Educ Today.

[16] Cohen D, Crabtree B. Lincoln, and Guba’s evaluative criteria. Qual Res Guidel. 2006; http://www.qualres.org/HomeLinc-3684.html. Accessed 31 May 2020.

[17] Kackin O, Ciydem E, Aci OS, Kutlu FY (2020). Experiences and psychosocial problems of nurses caring for patients diagnosed with COVID-19 in Turkey: a qualitative study. Int J Soc Psychiatr.

[18] Liu O (2020). The experiences of health-care providers during the COVID-19 crisis in China: a qualitative study. Lancet Glob Health.

[19] Explaining the experiences, challenges, and adaptation strategies of nurses in caring for patients with COVID-19: a qualitative study in Iran. Available from: https://www.readcube.com/articles/10.21203%2Frs.3.rs-100575%2Fv1. Accessed 31 Oct 2020. 10.1186/s12912-022-00937-8 PMC9238071 35765051

[20] Karami Z (2020). The lived experience of nurses caring for patients with COVID-19 in Iran: a phenomenological study. Risk Manag Healthc Policy.

[21] Sun N, Wei L, Shi S, Jiao D, Song R, Ma L, Wang H, Wang C, Wang Z, You Y, Liu S, Wang H (2020). A qualitative study on the psychological experience of caregivers of COVID-19 patients. Am J Infect Control.

[22] Wu C (2019). Identifying the positive energy for retention in clinical nurses: A focus group study. J Nurs Manag.

[23] Zhang Y, Wei L, Li H, Pan Y, Wang J, Li Q, Wu Q, Wei H (2020). The psychological change process of frontline nurses caring for patients with COVID-19 during its outbreak. Issues Mental Health Nurs.

[24] Chvance A (2020). Ensuring mental health care during the SARS-CoV-2 epidemic in France: a narrative review. L’encephale.

[25] Jamison C, et al. Reflections on nursing ingenuity during the COVID-19 pandemic. J Neurosci Nurs. 2020. 10.1097/JNN.0000000000000525 PMC7172973 32221059

[26] Kim Y (2018). Nurses' experiences of care for patients with the Middle East respiratory syndrome-COVID-19 in South Korea. Am J Infect Control.

[27] Xiang Y. T, et al. Timely mental health care for the 2019 novel COVID-19 outbreak is urgently needed. Lancet Psychiatr. 2020;7.3:228–229. 10.1016/S2215-0366(20)30046-8. 10.1016/S2215-0366(20)30046-8 PMC7128153 32032543

[28] Taylor S, Landry CA, Rachor GS, Paluszek MM, Asmundson GJG (2020). Fear and avoidance of healthcare workers: an important, under-recognized form of stigmatization during the COVID-19 pandemic. J Anxiety Disord.

[29] Abulof U (2017). Introduction: why we need Maslow in the twenty-first century. Society.

[30] Catton H (2020). Nursing in the COVID-19 pandemic and beyond: protecting, saving, supporting, and honoring nurses. Int Nurs Rev.

[31] Jenna A (2021). Experiences of nurses during the COVID-19 pandemic: a mixed-methods study. AACN Adv Critical Care.

[32] Jing HE, et al. The experiences of nurses infected with COVID-19 in Wuhan, China: A qualitative study. J Nurs Manag. 2021. 10.1111/jonm.13256 PMC8013478 33480116

[33] Zhang Y, Wang C, Pan W, Zheng J, Gao J, Huang X, Cai S, Zhai Y, Latour JM, Zhu C (2020). Stress, burnout, and coping strategies of frontline nurses during the COVID-19 epidemic in Wuhan and Shanghai. China Frontiers Psychiatr.

